# DNA looping mediates nucleosome transfer

**DOI:** 10.1038/ncomms13337

**Published:** 2016-11-03

**Authors:** Lucy D. Brennan, Robert A. Forties, Smita S. Patel, Michelle D. Wang

**Affiliations:** 1 Department of Physics—Laboratory of Atomic and Solid State Physics, Cornell University, Ithaca, New York 14853, USA; 2 Howard Hughes Medical Institute, Cornell University, Ithaca, New York 14853, USA; 3 Department of Biochemistry and Molecular Biology, Rutgers-Robert Wood Johnson Medical School, Piscataway, New Jersey 08854, USA

## Abstract

Proper cell function requires preservation of the spatial organization of chromatin modifications. Maintenance of this epigenetic landscape necessitates the transfer of parental nucleosomes to newly replicated DNA, a process that is stringently regulated and intrinsically linked to replication fork dynamics. This creates a formidable setting from which to isolate the central mechanism of transfer. Here we utilized a minimal experimental system to track the fate of a single nucleosome following its displacement, and examined whether DNA mechanics itself, in the absence of any chaperones or assembly factors, may serve as a platform for the transfer process. We found that the nucleosome is passively transferred to available dsDNA as predicted by a simple physical model of DNA loop formation. These results demonstrate a fundamental role for DNA mechanics in mediating nucleosome transfer and preserving epigenetic integrity during replication.

## Abstract (web summary)

Replicating the genome presents a major challenge to the maintenance of the epigenetic landscape. Here the authors use a minimal experimental system to show how nucleosomes are transferred to double-stranded DNA, with the transfer mediated by a quantitative loop formation model.

At the replication fork, a complex interplay of proteins and DNA mediates the faithful duplication of DNA sequence and the subsequent packaging of nascent DNA into chromatin^1^,^2^. The spatial organization of epigenetic chromatin modifications is maintained throughout multiple rounds of cell division by the inheritance of parental nucleosomes and the ensuing duplication of covalent modifications to nascent nucleosomes^3^,^4^. Parental nucleosomes must be quickly moved from ahead of the progressing replisome to the newly replicated DNA behind it^4^,^5^,^6^, without loss of the histones H3/H4 tetramers into solution^7^,^8^. Thus, the transfer of parental nucleosomes requires stringent temporal and spatial regulation, and must remain reliable within a dynamic cellular environment. This alludes to a simplicity underlying the fundamental transfer mechanism—a means that is permissive of cellular changes and fluctuations, while still preserving the essential nucleosome topography.

Current views suggest that histone chaperones and nucleosome assembly factors act in a coordinated manner at the replication fork to shuttle parental histones and deposit nascent histones to newly replicated DNA^2^,^9^. However, this theory is only loosely described and neglects the role of DNA itself, the fundamental component of nucleosome organization and dynamics. The complex, dynamic environment within which nucleosome transfer occurs is a challenging setting to define essential roles of the individual components that underlie the transfer mechanism. To examine the role of DNA mechanics and identify the minimum system requirements for successful transfer, we utilized a DNA template with a single nucleosome and incrementally added complexity by first displacing the nucleosome mechanically, then with an isolated replicative helicase, and finally with a simplified replication complex. This process allowed us to quantify a fundamental aspect of nucleosome transfer and establish a critical role for the physical properties of DNA during chromatin replication.

## Results

### Passive nucleosome transfer after mechanical displacement

**Figure 1:**
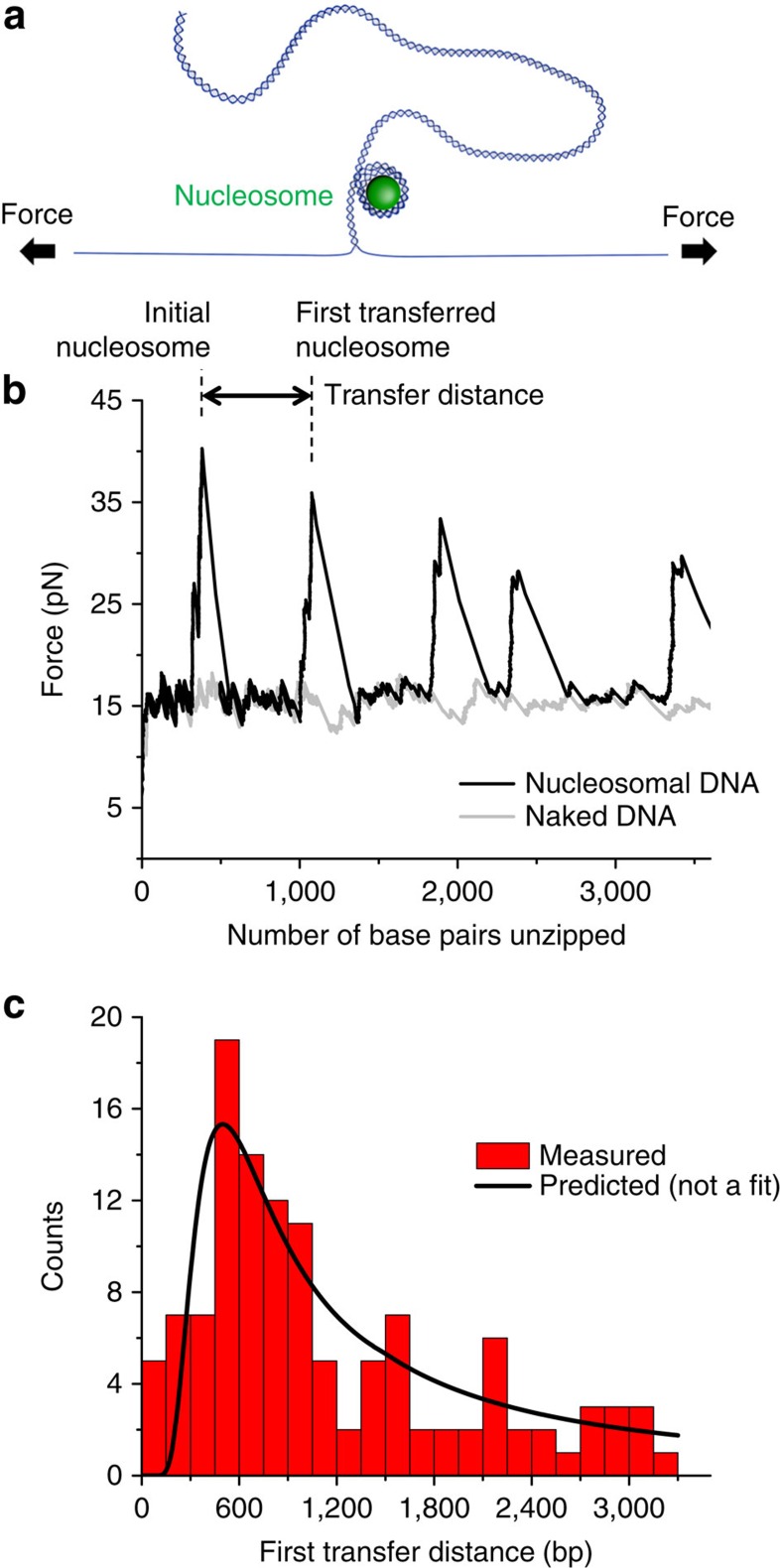
Mechanical displacement of a single nucleosome. (**a**) Experimental configuration. A single dsDNA molecule was mechanically unzipped using an optical trap (Supplementary Fig. 1A). The dsDNA contained a positioned nucleosome followed by a long naked DNA segment (Supplementary Fig. 2A, Supplementary Table 1 and Methods). (**b**) A representative unzipping trace. A force rise from the naked DNA baseline indicated the detection of a bound protein complex. A dashed vertical line indicates the dyad location of a nucleosome. *N*=121 traces. (**c**) Histogram of nucleosome transfer distance. A transfer distance was obtained from the first transfer event of each trace. The histogram was obtained by pooling data from 121 traces. The prediction (not a fit) from the DNA looping model is plotted for comparison. The resulting Pearson test gives a reduced *χ*^2^ of 0.53 with a *P* value of 0.81 (Methods; Supplementary Fig. 4A).

In a solution with no free histones, a double-stranded DNA (dsDNA) template, containing a single positioned nucleosome, was mechanically unzipped using an optical trap (Fig. 1a, Supplementary Figs 1A and 2A, and Supplementary Tables 1 and 2). The resulting unzipping force served as a sensitive detector for the presence of the nucleosome, with a force rise above the naked DNA baseline indicating both nucleosome location and composition (Fig. 1b)^10^,^11^,^12^,^13^,^14^,^15^,^16^. During unzipping, the force first followed that of naked DNA, until the fork reached the positioning sequence, and then a marked force rise characteristic of a canonical nucleosome^11^ occurred, followed by a force drop. As unzipping continued, additional distinct force signatures emerged along the downstream DNA (in front of the moving fork), indicating a re-association of the displaced histone. When the same construct was unzipped in the opposite direction, no force rise occurred until the nucleosome-positioning sequence, illustrating the mono-nucleosome nature of the template (Supplementary Fig. 3). We therefore attribute the subsequent force signatures to the transfer of the original nucleosome. The vast majority of unzipping traces (99%) showed at least one transfer event. For the first such transfer event on each unzipped template (Supplementary Table 2), 67% of traces showed a force signature consistent with that of a nucleosome^10^,^11^,^12^,^13^,^14^,^15^,^16^ and 32% were consistent with that of a tetrasome^10^,^13^, though it is possible that some of these may have been hexasomes^17^,^18^. These results are in agreement with previous findings that parental H3/H4 tetrasomes generally remain intact after replication fork passage, while H2A/H2B dimers are more labile^19^,^20^,^21^.

### Nucleosome transfer is consistent with DNA loop formation

Nucleosome transfer in this experimental system could occur via a diffusion-based process, during which histones dissociate from the DNA after being displaced by the fork, diffuse in solution, and then re-associate with another DNA segment. However, this mechanism would result in a distribution of transfer distance that peaks at zero, because histones would most likely associate with a DNA segment in close proximity, such that short distance transfer dominates. In contrast, our data of the first transfer distance peaked at 500–700 bp, and do not support histone dissociation and diffusion. Previous studies also provide evidence indicating that during replication, parental histones are not released into solution^7^,^8^,^22^, arguing against a diffusion-based mechanism.

An alternative mechanism for nucleosome transfer is based on DNA looping. On fork invasion of the nucleosome, the histone surfaces become partially exposed and available dsDNA may loop back onto the histone surfaces and capture the histones. As the fork progresses, the nucleosome is thereby repositioned to another location on the DNA. Although this possibility was raised nearly 20 years ago^8^, there has been no direct experimental evidence to date. Importantly, support for such a model must be made quantitatively, because DNA loop formation makes explicit predictions on the loop size distribution^23^,^24^,^25^,^26^ and thus the nucleosome transfer distance distribution. Transfer distances below 200 bp are energetically unfavourable as the persistence length of DNA is ∼150 bp, thus prohibiting the formation of small loops. Very long transfer distances are also improbable, because the putative acceptor DNA must sample a large volume, reducing its chance of encountering the initial nucleosome. Consequently, the defining features of the DNA looping model are an extremely low transfer probability at short distances, a sharp rise in the probability at ∼200 bp followed by a peak at ∼500 bp and a long tail.

Figure 1c shows a comparison of measured transfer distances and a direct prediction by the loop formation theory (not a fit). There is a good agreement between the two distributions (Methods; Supplementary Fig. 4A). The loop formation model depends predominantly on the persistence length of DNA, which dictates the likelihood of downstream DNA being in close proximity to the nucleosome. Although persistence length is DNA sequence-dependent^27^, such dependence should be secondary on the length scale considered here. Consistent with this, an additional experiment conducted with a DNA template of a different sequence yielded a similar transfer distance distribution (Supplementary Fig. 5). In addition, we found that increasing the rate of unzipping 10-fold does not lead to major changes in the transfer distance distribution or efficiency, although there is a slight increase in the nucleosome fraction (Supplementary Fig. 6 and Supplementary Table 2b).

### Local DNA concentration and DNA elasticity dictate transfer

**Figure 2:**
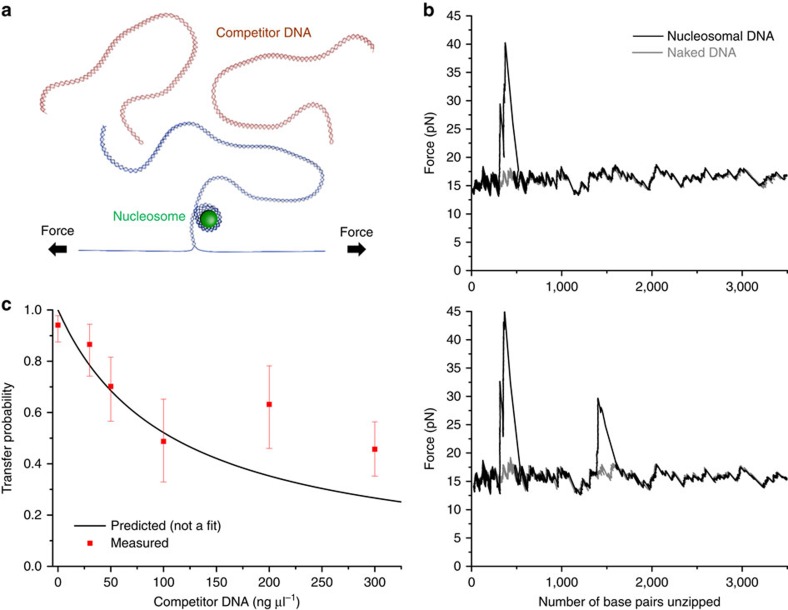
Mechanical displacement of a single nucleosome in the presence of competitor DNA. (**a**) Experimental configuration. A single dsDNA molecule was mechanically unzipped using an optical trap (Supplementary Fig. 1A). The dsDNA contained a positioned nucleosome followed by a long naked DNA segment (Supplementary Fig. 2A, Supplementary Table 1 and Methods). Linear competitor dsDNA of 2,987 bp was introduced into the chamber at varying concentrations immediately before mechanical disruption. *N*=121, 52, 57, 39, 38 and 92 traces for 0, 30, 50, 100, 200 and 300 ng μl^−1^ competitor DNA concentrations, respectively. (**b**) Two example unzipping traces in the presence of 100 ng μl^−1^ of competitor DNA. The top trace shows an absence of a transferred nucleosome to the downstream DNA, whereas the bottom trace shows the presence of a transferred nucleosome. (**c**) The probability of nucleosome transfer to downstream dsDNA as a function of competitor DNA concentration. Error bars represent 95% confidence intervals (Methods). A direct prediction (not a fit) based on DNA looping and a simple competitive binding relation (Methods) is shown for comparison.

To further examine whether DNA looping mediates nucleosome transfer, we carried out nucleosome disruption experiments in the presence of competitor DNA (Fig. 2a), which should compete with the downstream DNA for acceptance of a transferred nucleosome. The DNA looping model directly predicts an effective local concentration of the available downstream DNA at the nucleosome (Methods), and thus nucleosome transfer to downstream DNA should decrease with an increase in competitor DNA concentration, following a simple competitive binding relation (Methods).

In these experiments, nucleosomes were disrupted in the presence of varying concentrations of competitor DNA that was of nearly equal length as that of the downstream DNA. As expected, with an increase in competitor DNA concentration, the probability for nucleosome transfer to the downstream DNA decreased. Example trace without and with transfer to the downstream DNA are shown in Fig. 2b. Figure 2c shows a summary of the probability of transfer to the downstream DNA as a function of competitor DNA concentration, along with a direct prediction (not a fit) based on a simple competitive binding relation. There is good agreement between measurements and prediction at competitor DNA concentrations ≤100 ng μl^−1^; concentrations above this threshold resulted in measured values somewhat larger than predicted. Deviation in this range is likely due to the use of a simple competitive binding relation without consideration of the excluded volume effect (Methods), which becomes significant at high competitor DNA concentrations resulting in a preference for intra-DNA transfer. Therefore, over the range where the competitive binding relation holds, these results support DNA loop formation as the mechanism of nucleosome transfer.

### Helicase-induced nucleosome transfer

**Figure 3:**
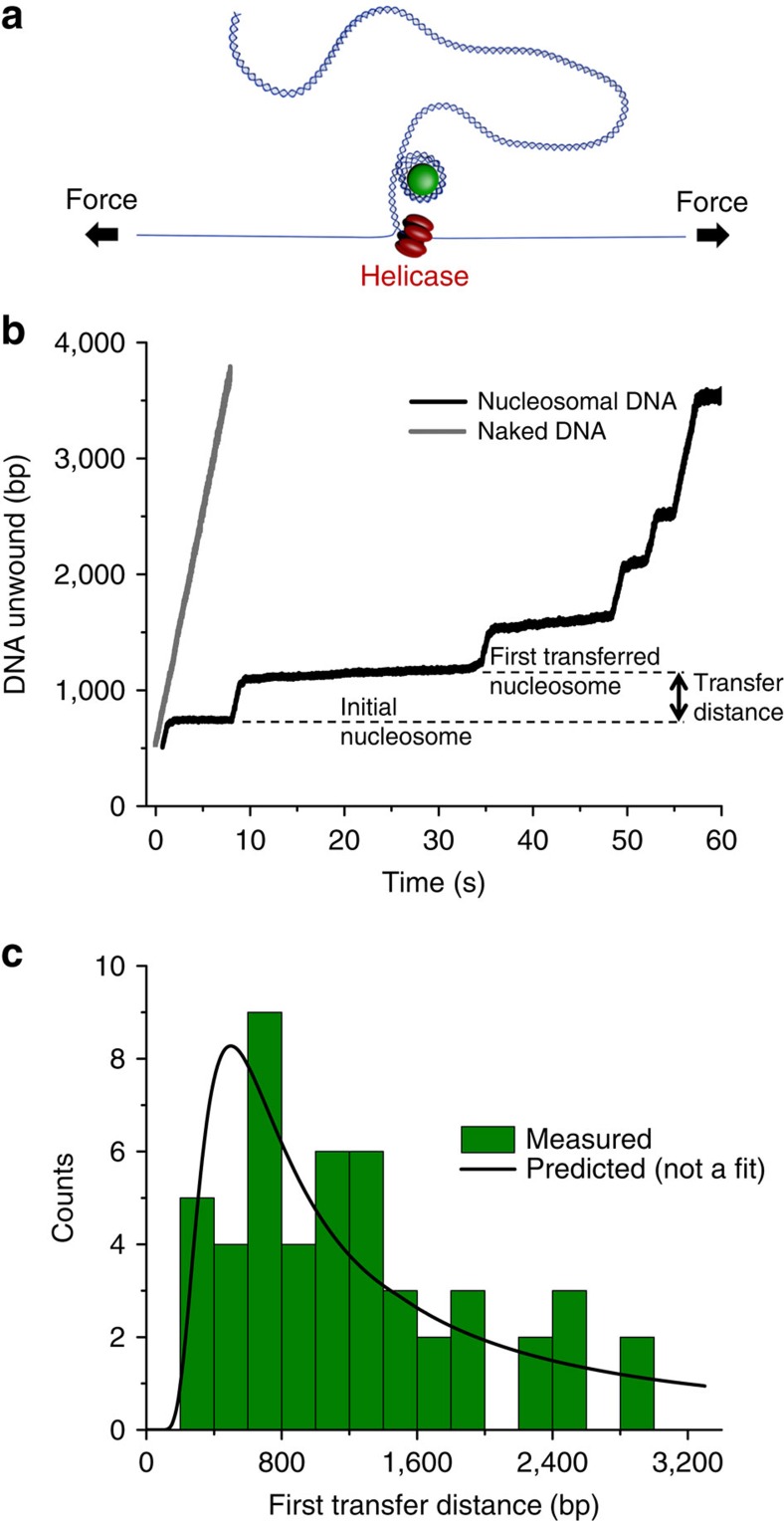
Helicase displacement of a single nucleosome. (**a**) Experimental configuration. A single dsDNA molecule was unwound by a T7 helicase as the two strands of the DNA were held under 12 pN of force by an optical trap, which assisted helicase unwinding but was insufficient to mechanically separate the dsDNA (Supplementary Fig. 1B). The nucleosomal DNA template is specified in Supplementary Fig. 2A, Supplementary Table 1 and Methods. (**b**) Representative helicase-unwinding traces on a nucleosomal (black) or naked (grey) template. Helicase unwinding was interrupted by discrete pauses along the DNA template. Dashed lines indicate the dyad locations of the initial positioned nucleosome and the transferred nucleosome. *N*=49 traces. (**c**) Histogram of nucleosome transfer distance. A transfer distance was obtained from the first transfer event of each trace as indicated by the arrow in Fig. 2b. The histogram was obtained by pooling data from 49 traces. The prediction (not a fit) from the DNA looping model is plotted for comparison. The resulting Pearson test gives a reduced *χ*^2^ of 0.31 with a *P* value of 0.95 (Methods; Supplementary Fig. 4A).

Although fork progression was initially carried out mechanically, *in vivo* it is mediated by helicases that unwind dsDNA during replication. We therefore used T7 helicase as a simple model system to investigate the fate of a single nucleosome located on a parental dsDNA template during unwinding (Fig. 3a, Supplementary Figs 1B and 2A, and Supplementary Table 1). As shown in Fig. 3b, before encountering the nucleosome, the helicase unwound the dsDNA at the expected rate, as reported previously^28^,^29^,^30^,^31^,^32^. On encountering the nucleosome, the helicase showed a discrete pause, consistent with previous studies that characterized nucleosomes as major barriers for helicase unwinding^33^. Initial pausing occurred near the dyad region of the positioned nucleosome, which contains the strongest histone–DNA interactions^10^,^11^,^12^,^15^,^16^. In 89% of the traces, helicase eventually exited the pause within the experimental time window of 150 s, and then proceeded at its initial speed, indicating the complete displacement of the nucleosome. As the helicase unwound further along the DNA, it paused again at locations initially lacking nucleosomes. These additional pauses suggest nucleosome transfer downstream from its original location. Analysis of the distance of the first transfer event revealed a distribution that was again in agreement with prediction by the DNA loop formation model (Methods; Supplementary Fig. 4B). Thus, a simple passive mechanism is able to account for nucleosome transfer during fork progression, carried out either mechanically or by a motor protein.

### Nucleosome transfer to leading strand at replication fork

**Figure 4:**
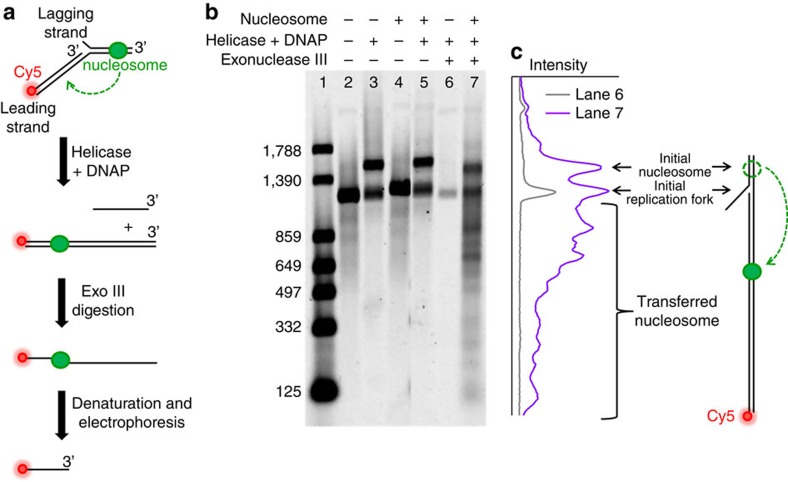
Replisome displacement of a single nucleosome. (**a**) Experimental configuration. Leading strand replication was carried out using the T7 replisome on a Cy5-labelled parental template containing a single nucleosome with minimal dsDNA downstream. (**b**) Nucleosome transfer after replication. The replication product was exonuclease III-digested and assayed on a denaturing gel (lane 7). Lane 1 is a ladder and lanes 2–6 are control experiments. Lanes digested with exonuclease III were loaded with five times as much sample as the other lanes to achieve more accurate quantification. *N*=4 replicates (Supplementary Fig. 7). (**c**) A line scan of lane 7 contained contributions from both the transferred nucleosome as well as background. In particular, a fraction of replisomes did not proceed past the nucleosome, and another fraction contained inactive replisome bound at the initial fork (see lane 6). The background in lane 6 is removed from lane 7 during subsequent analysis. The template schematic right of the line scan explains some features of the band positions in the gel and the line scans.

During DNA replication, dsDNA available for nucleosome transfer is located behind the replication fork on the nascent daughter duplexes, which are poised to accept parental nucleosomes from ahead of the replication fork^22^,^34^,^35^. We hypothesize that if nucleosome transfer is dictated by DNA loop formation, transfer should take place on the upstream dsDNA, in a similar manner as demonstrated for the downstream dsDNA. To investigate this hypothesis, we carried out leading strand replication using the T7 replisome to generate upstream dsDNA. The parental DNA template contained a single nucleosome with minimal naked DNA downstream (ahead) of the nucleosome (Fig. 4a, Supplementary Fig. 2B and Supplementary Table 1). To quantitatively assay the position of the transferred nucleosome, the 5′-end of the replicated leading strand was fluorescently labelled, and the replication product was subjected to exonuclease III digestion before being assayed by a denaturing gel (Fig. 4b, replicates shown in Supplementary Fig. 7). The single-stranded DNA (ssDNA) resistant to digestion provided a quantitative measure for nucleosome position following DNA replication. The resulting distribution of the ssDNA lengths shows nucleosome transfer, peaked at 500–700 bp upstream of the initial nucleosome position. Although the measured transfer distance showed some sequence preference not accounted for by the loop formation model in its current simplest form, the overall features of the distribution are consistent with the model. Furthermore, the measured effect of competitor DNA on nucleosome transfer in these bulk replication assays is again well predicted by the DNA looping theory (Supplementary Fig. 8).

## Discussion

**Figure 5:**
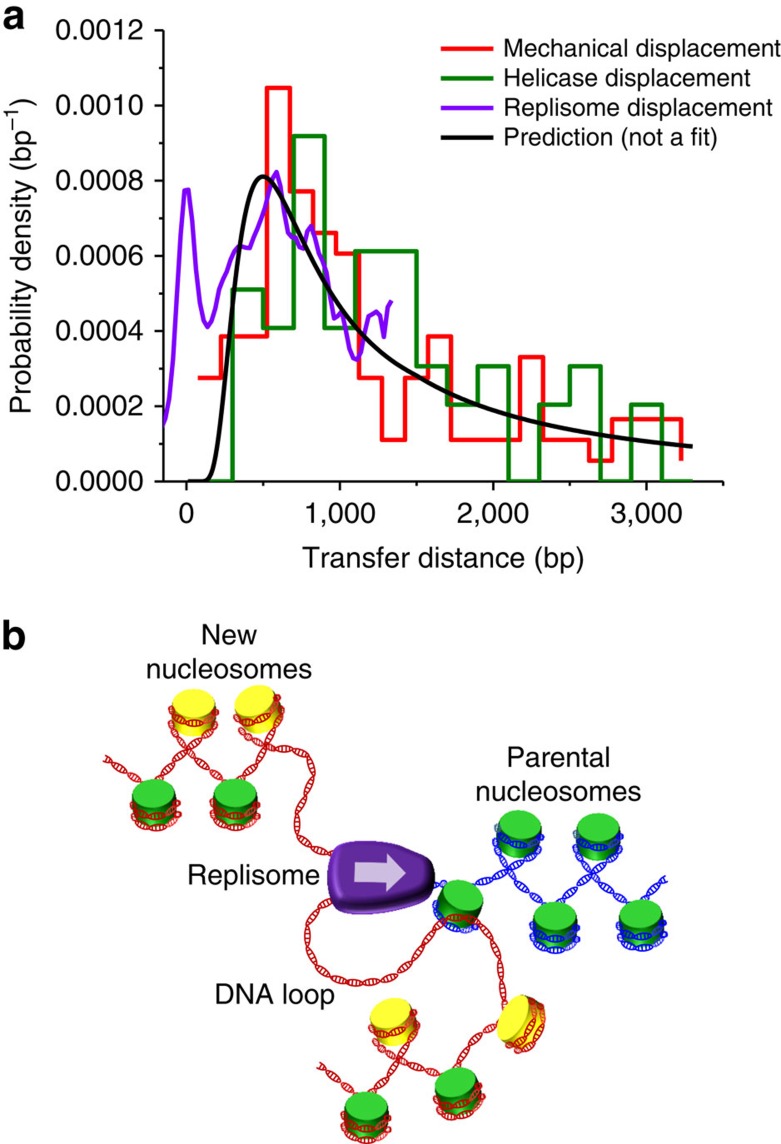
The passive nucleosome transfer model via DNA loop formation. (**a**) Comparison of nucleosome transfer distance distributions as measured using three experimental approaches: mechanical fork progression (red; Fig. 1c); helicase unwinding (green; Fig. 2c); and leading strand replication (purple; Supplementary Fig. 7). Note that the peak near zero from the leading strand replication curve (purple) was background introduced by the fraction of reaction that did not proceed past the nucleosome as indicated in Fig. 3. The prediction (black, not a fit) from the DNA looping model is also shown for comparison. (**b**) A mechanistic model of passive nucleosome transfer mediated by DNA loop formation. When a replisome (purple) encounters a parental nucleosome (green) at the replication fork, a DNA loop forms in one of the daughter duplexes (red), bridging the nucleosome from its initial location to its new location and thus facilitating direct transfer to the daughter duplex. Nascent histones (yellow) are also deposited on the daughter strands by chaperones.

Taken together, results from these three distinct experimental approaches provide consistent support for passive nucleosome transfer by DNA loop formation (Fig. 5a). As a nucleosome is displaced, it will be spontaneously transferred to available dsDNA, and this transfer is mediated by the formation of a DNA loop that bridges the nucleosome from its initial location to its new location (Fig. 5b). Previous studies found nucleosomes remain associated with DNA during transcription after the passage of RNA polymerase^36^,^37^. Earlier studies with pol III suggested a loop of 80 bp (ref. 37); whereas more recent work with pol II favours a ‘zero-size' DNA loop^38^. In contrast, for DNA replication, such small, or non-existent, loops are not consistent with previously measured *in vivo* distance scales^34^,^35^,^39^.

Indeed, a number of *in vivo* and *in vitro* chromatin replication studies support key aspects of our looping model. DNA loop formation requires at least ∼200 bp of free dsDNA to form a minimal DNA loop^26^, consistent with the 200–600 bp of available naked nascent dsDNA present immediately upstream of the replication fork *in vivo*^22^,^34^,^35^. In addition, parental nucleosomes have been shown to be located within ∼400 bp of their original positions after the completion of the cell cycle^7^,^35^,^39^, close to the most probable loop size. DNA loop formation may also be facilitated by the configuration^40^ of nascent dsDNA strands as they emerge from the replisome, which would contribute to the partitioning^19^,^41^ of nucleosomes between the two daughter strands by coordinating nucleosome transfer with DNA synthesis^42^.

*In vivo*, passive transfer would only occur if there is sufficient available dsDNA to accept parental histones. Consistent with this requirement, overexpression of new histones or perturbation of chaperone function both result in replication fork stalling^9^. This implies that when daughter DNA is saturated with new histones, or new histones are not positioned properly, the parental nucleosome at the fork cannot be efficiently transferred and therefore becomes a substantial barrier for replication. Nascent histone deposition is likely coordinated with the transfer of parental nucleosomes, possibly by regulation of the deposition of new histones through a feedback mechanism involving the transfer of parental nucleosomes^6^,^43^,^44^.

Although our model does not require specific interactions of histones with the replisome, recent studies have shown that histone H3 may interact with the eukaryotic helicase^45^, providing insight into how replisome progression and histone dynamics may be coordinated^42^. However, the action by which this potential intermediate transfers parental histones to the nascent DNA has yet to be elucidated and is still controversial^46^. The *in vivo* mechanism for nucleosome inheritance likely requires the coordination of many factors acting at, and around, the replication fork. These complex processes can take place on a simple platform dictated by DNA mechanics. The data presented here have quantified the ability of available DNA to facilitate the transfer of parental nucleosomes.

Our proposed model of passive parental nucleosome transfer via DNA loop formation describes a fundamental mechanism to facilitate parental nucleosome transfer while also permitting broader coordination for the deposition of new histones. DNA loop formation thus provides a simple pathway that facilitates cellular complexity by exploiting fundamental physical properties.

## Methods

### Protein purification

Histones were purified using hydroxyapatite precipitation from HeLa-S3 cells purchased from the National Cell Culture Center^10^,^11^,^12^,^16^,^20^. Nuclei were extracted from a pellet from 6 l of cells in Nuclear Pellet Prep Lysis Buffer (20 mM HEPES (pH 7.5), 3 mM MgCl_2_, 250 mM sucrose, 0.5% (v/v) IGEPAL CA-630 (NP-40) nonionic detergent, 1 tablet per 50 ml Complete protease inhibitor cocktail (Roche) and 3 mM 2-mercaptoethanol)^47^. The nuclei pellets were frozen in liquid nitrogen and stored at −80 °C. Core histones were purified using a hydroxyapatite Bio-gel HTP gel (Bio-Rad Laboratories) slurry, according to methods by Wolffe and Ura^48^, with the omission of MNase digestion before fractionation. Aliquots of purified histones were stored in −80 °C at a final concentration of 2.7 μM.

Nucleosomes were assembled on the Widom 601 nucleosome-positioning element^49^ by salt dialysis^10^,^11^,^12^,^13^,^15^,^16^,^20^,^50^,^51^,^52^,^53^. For the mechanical displacement assay, nucleosomes were assembled on a 764 bp template at a molar ratio of 1.25:1.00 of histone octamer to DNA. For the helicase displacement assay, nucleosomes were assembled on an 896 bp template in the presence of salmon sperm competitor DNA (Life Technologies) at a 1.5:1.0:4.0 molar ratio of histone octamer:template DNA:salmon sperm DNA. For the replication gel experiments, nucleosomes were assembled on a 250 bp template at a molar ratio of 1.75:1.00 of histone octamer to DNA. Template details can be found in Supplementary Table 1 and example native gels of nucleosome assembly can be found in Supplementary Fig. 9.

Wild-type T7 helicase gp4A' was purified from *Escherichi coli*. by the Patel Lab^54^.

### DNA template construction

All experiments required the use of forked DNA templates, each of which consisted of two arms and a trunk. These forked DNA structures were prepared by the ligation of DNA adapter oligos (Supplementary Table 1) to a labelled PCR product. The adapter oligos contained a region that annealed to one another thus forming a Y-structure DNA template^55^. For all single-molecule experiments, each arm was ∼1,000 bp (see Supplementary Table 1 for sequences of all DNA segments). For the mechanical displacement experiments, the trunk consisted of either a 764 or a 896 bp segment containing the 601 nucleosome positioning sequence, which started at 298 or 596 bp, respectively, from the initial fork. This segment was then ligated to a 2,987 bp downstream DNA segment at 16 °C for 90 min. This produced a template with 3,483 bp (or 3,258 bp) of DNA downstream of the start of the initial positioned nucleosome. For Supplementary Fig. 5 on the effect of DNA sequence on nucleosome transfer, the 764 bp segment was ligated to a 2,927 bp segment, which was identical to the 2,987 bp segment except for its slightly shorter length, and a reversed sequence. The helicase displacement experiments were performed using only the template containing the 896 bp segment ligated to the 2,987 bp downstream DNA segment. The 2,987 bp segment was also used as the competitor DNA in Fig. 2 and Supplementary Fig. 8.

For the leading strand replication gel experiments, the initial replication fork consisted of a trunk of a 298 bp dsDNA containing the 601 nucleosome-positioning element, a 30-nucleotide flap of ssDNA to facilitate T7 helicase loading to the replication fork^29^,^31^,^56^, and a 1,189 bp dsDNA leading strand.

### Mechanical displacement of nucleosome assay

DNA tethers were formed in flow chambers and were unzipped using an optical trap with a loading rate clamp of 10 pN s^−1^ (Fig. 1 and Supplementary Figs 3 and 5) through the bound protein^10^,^11^,^12^,^15^,^16^,^28^,^29^,^30^,^31^,^55^,^57^,^58^,^59^,^60^. Briefly, chambers were first incubated with anti-digoxygenin at 0.2 mg ml^−1^ for 5 min, and then the surface was blocked by incubation with casein at 5 mg ml^−1^ for 5 min. Then DNA templates were flowed into the chamber at 100 pM for 5 min. This was followed by incubation with 500 nm streptavidin-coated microspheres at 4 pM, which bound to the biotin linkers on the DNA template. Finally, chambers were washed with nucleosome-unzipping buffer (10 mM Tris (pH 8), 1 mM EDTA, 100 mM NaCl, 3% (v/v) glycerol,1.5 mM MgCl_2_, 1 mM dithiothreitol (DTT), 0.02% Tween 20, 2 mg ml^−1^ AcBSA (Ambion) and 0.1 mg ml^−1^ casein). Experiments were performed at room temperature (24 °C). For experiments involving competitor DNA, the competitor DNA was diluted in 1 × nucleosome-unzipping buffer to the specified concentration and then flowed into the single-molecule chamber immediately before data acquisition.

The number of DNA base pairs unzipped as at each time point was calculated from the raw force and extension measurements^59^,^61^,^62^. We then applied an algorithm that defined peaks as a force rise ≥20 pN, which increased at a rate >3 pN s^−1^. Nucleosomes were identified as containing peaks separated by <65 bp, up to a maximum of three, in accordance with previous work^11^. Individual force peaks separated by more than 65 bp were considered to be tetrasomes^10^,^13^. Nucleosome transfer distances were measured as the distance from the first peak within the 601 nucleosome-positioning element to the first peak outside of the defined nucleosome force signature. For a trace to be categorized as having no nucleosome transfer, it must show no detectable force rise significantly above the naked DNA unzipping baseline. Only tethers that were unzipped to the end of the DNA construct were included in the data set to avoid any sampling bias due to nicks in the DNA.

### Helicase displacement of nucleosome assay

DNA tethers were prepared as described above, and helicase preparation was as described below^28^,^30^,^31^. Briefly, 1.5 nM of the helicase monomer was incubated for up to 20 min in the modified nucleosome-unzipping buffer (10 mM Tris (pH 8), 1 mM EDTA, 100 mM NaCl, 3% (v/v) glycerol,1.5 mM MgCl_2_, 1 mM DTT, 0.02% Tween 20, 2 mg ml^−1^ AcBSA (Ambion), 1 mg ml^−1^ casein and 2 mM dTTP). Magnesium chloride was added to a final concentration of 3 mM immediately before helicase addition to the sample chamber. Experiments were conducted using the following steps. First, ∼400 bp of dsDNA were mechanically unzipped, at a constant velocity of 200 nm s^−1^ for 2 s, to produce a ssDNA-loading region for helicase. The tether was held at a constant position for up to 120 s for helicase loading to occur, if loading did not occur within this time frame, the tether was released and a new tether was selected. If the force dropped below 10 pN, owing to helicase loading and initiation of unwinding, the tether was then held at a constant force of 12 pN as the helicase position was tracked.

To detect helicase pausing, the dwell time of each trace as a function of the number of base pairs unzipped was calculated, with a bin size of 10 bp. We then defined dwell times of at least 0.5 s per 10 bp bin as pauses. The end of a pause was defined as dwells <0.2 s per 10 bp bin. Pauses separated by more than 30 bp were considered to be spatially distinct events. To determine the nucleosome transfer distance, we measured the number of base pairs between the first pause, which was located within the initial positioned nucleosome element, and the second pause. As was done for the mechanical displacement experiments, only tethers that were unwound to the end of the DNA construct were included in the data set.

### Bulk DNA replication assay

T7 replication assays were performed in 50 μl reaction volumes (or 10 μl reaction volumes for controls, which were not exonuclease III digested, as indicated in the main text) containing 40 mM Tris (pH 7.5), 10 mM MgCl_2_,10 mM DTT, 0.1 mg ml^−1^ AcBSA (Ambion) and 0.6 mM of each of dATP, dCTP, dGTP and dCTP (Roche). DNA templates were added to a final concentration of 10 nM. Replisomes were formed by pre-incubating 1 unit per μl T7 DNA polymerase (NEB) and 1 μM T7 helicase in reaction buffer on ice for 5 min, and then were added to a final concentration of 0.1 unit per μl T7 DNA polymerase (NEB) and 100 nM T7 helicase and incubated at 37 °C for 10 min. Samples were then buffer exchanged into 1 × NEBuffer 1 (NEB) using Amicon Ultra-0.5 centrifugal filter units with Ultracel-30 membranes (Millipore). A volume of 400 μl 1 × NEBuffer 1 was added and samples were centrifuged at 5,000*g* for 5 min at 4 °C four times total. Samples were spun an additional 5 min at 4 °C for the final concentration to reduce the retained volume. Each reaction was then digested with 100 units of exonuclease III (NEB) at 37 °C for 30 min.

Samples were precipitated by addition of 500 μl solution containing 0.5% linear polyacrylamide, 7% saturated ammonium acetate and 91% ethanol^63^, incubated overnight at −80 °C and centrifuged at 16,000*g* for 20 min. Samples were then decanted and 500 μl 70% ethanol was added followed by vigorous vortex mixing. This was followed by centrifugation at 16,000*g* for 10 min. Tubes were then carefully decanted, and dried for 5 min in a vacuum chamber. Pellets were resuspended in 20 μl of alkaline gel buffer (5 mM NaOH and 1 mM EDTA) by vortexing and incubated for 30 min at 37 °C. A volume of 4 μl alkaline loading buffer (50% glycerol, 30 mM NaOH and 6 mM EDTA) was then added and each sample was heated at 95 °C for 5 min to denature the DNA, and then placed on ice. Samples were separated on 1% agarose gels in alkaline gel buffer using electrophoresis at 4.7 V cm^−1^ for 4 h, and quantified using a Typhoon imager (GE).

Data were then converted from intensity at each position within a gel scan to probability distributions for nucleosome transfer distance. The Cy5-labelled DNA ladder was created using PCR products of 125, 332, 497, 649, 859, 1,390 and 1,788 bp. We calculated the DNA length corresponding to these ladder positions by fitting the log of ladder band length versus gel position to a quadratic function, and then linearly interpolated between ladders run in different lanes. The intensity was summed within each lane in 25 bp increments to obtain a probability distribution versus DNA length. Transfer distance distributions were obtained by subtracting the measured DNA length from the known initial position of the 601 sequence (1,500 bp for the Cy5-labelled strand). Data obtained for naked DNA templates were subtracted from data for nucleosome transfer to account for incomplete digestion by exonuclease III.

For replication reactions run in the presence of competitor DNA (Supplementary Fig. 8), the data were analysed as described above with the following modifications. The gel scan from each reaction was first background subtracted and then normalized by the total amount of fully replicated DNA before exonuclease digestion. The normalized nucleosome transfer was then calculated by integrating the line scans from 1,200 to 100 bp (corresponding to 300–1,400 bp transfer distances) and normalizing it by that under 0 ng μl^−1^ competitor DNA condition.

### DNA loop formation modelling

The DNA looping probability, or the Jacobson–Stockmayer *J*-factor^64^, was calculated for a dsDNA with a persistence length of 50 nm (refs 62, 65) using the worm-like-chain model from equation (50) of Shimada and Yamakawa^66^. This formula diverges for DNA lengths longer than 2,000 bp, so for long DNA we applied the Daniels approximation^67^. For a given length of DNA, this *J*-factor gives the effective DNA molar concentration of one end of a DNA molecule at its other end under the assumption that the DNA ends are free to adopt any orientation relative to one another. Therefore, the *J*-factor describes the equilibrium constant for forming a DNA loop. To compare with our measured nucleosome transfer distance distribution, the *J-*factor is converted to a probability density function over all possible lengths and then rescaled to the number of traces in each data set. This comparison assumes that the probability of a nucleosome transfer to a destination site is proportional to the equilibrium probability of DNA loop formation that bridges the nucleosome's original position with the destination site. This is a reasonable assumption since in our unzipping configuration, the timescale of a nucleosome transfer (estimated based on the time it takes to unzip through a nucleosome: about 4 s for Fig. 1 and 0.44 s for Supplementary Fig. 6) is much slower than that of the mean first passage time of DNA looping, which is in the order of milliseconds^68^.

To determine the effective DNA weight concentration of the available downstream DNA at the nucleosome (*C*_0_), the *J*-factor in molar concentration for a given length of DNA was then integrated over the entire length of the downstream DNA before conversion to a weight concentration. For the 2,987 bp downstream DNA, this corresponds to *C*_0_=100 ng μl^−1^. In the presence of competitor DNA, a simple competitive binding relation was used to predict the probability of transfer to the downstream DNA as a function of competitor DNA concentration (*C*): 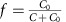. This expression does not consider any excluded volume effect, which should become substantial at high competitor DNA concentrations. This should occur when the volume explored by a single competitor DNA molecule over its radius of gyration reaches the mean volume available for each competitor DNA molecule in solution. We estimate that for the 2,987 bp DNA, the excluded volume effect needs to be considered for *C*>110 ng μl^−1^.

### Statistical analysis

To compare the measured transfer distributions with the DNA looping model, we carried out the Pearson's reduced *χ*^2^-test to quantitatively determine the goodness of the agreement for the data shown in both Figs 1c and 3c. Both data sets were binned into 400 bp bins as was the theory curve. In each case, this test yielded a reduced *χ*^2^-value and a corresponding *P* value. The *P* value is the probability of observing a difference between the measurements and theory as extreme as what have been measured, assuming the measurements were from the theoretical distribution. The *P* values (0.81 for Fig. 1c and 0.95 for Fig. 3c) indicate a strong agreement between the measurements and the DNA looping model.

Error bars in Fig. 2c and Supplementary Table 2 were calculated using Matlab's built-in function, binofit. [phat,pci]=binofit(*x*,*n*) returns a maximum likelihood estimate of the probability of success, phat, in a given binomial trial based on the number of successes, *x*, observed in *n* independent trials and the 95% confidence intervals, pci. binofit uses the Clopper–Pearson method to calculate confidence intervals.

### Data availability

DNA sequences, plasmids and DNA looping theory calculations from this study are available on request from the corresponding author.

## Additional information

**How to cite this article:** Brennan, L. D. *et al*. DNA looping mediates nucleosome transfer. *Nat. Commun.* **7**, 13337 doi: 10.1038/ncomms13337 (2016).

**Publisher's note:** Springer Nature remains neutral with regard to jurisdictional claims in published maps and institutional affiliations.

## Supplementary Material

Supplementary Information Supplementary Figures 1-9, Supplementary Tables 1-2 and Supplementary References
