# Supplementary material for: DNA looping mediates nucleosome transfer
Source: Nat Commun. 2016 Nov 3;7:13337. doi: 10.1038/ncomms13337 (PMC5097161; doi:10.1038/ncomms13337)
Supplement: Supplementary Information — Supplementary Figures 1-9, Supplementary Tables 1-2 and Supplementary References [file ncomms13337-s1.pdf]

Supplementary Information

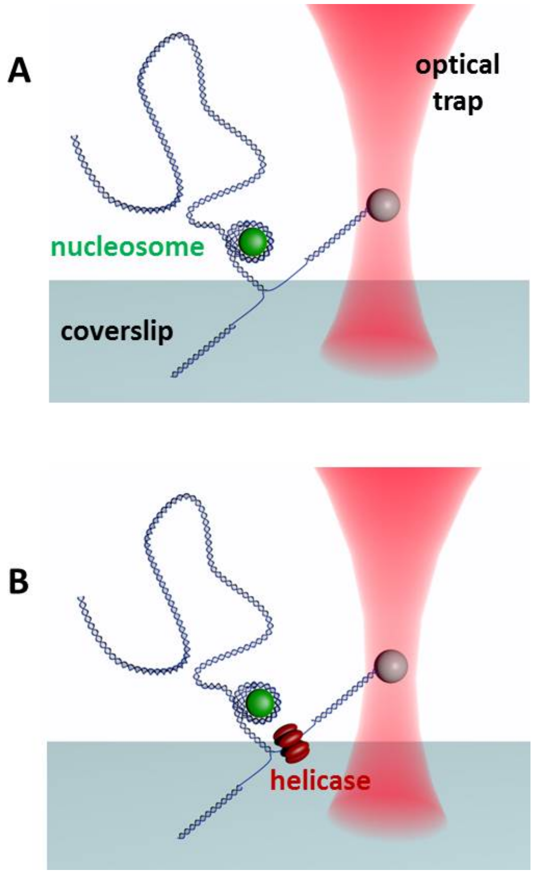

Supplementary Figure 1: Single molecule experimental configurations.

(A) Configuration for mechanical displacement of a nucleosome. A nucleosome was located on a dsDNA template to be mechanically unzipped. One strand of the template was anchored to the surface of a glass coverslip via a digoxygenin and anti-digoxygenin connection, and the other strand was attached via a biotin-streptavidin connection to a 500 nm polystyrene microsphere held in an optical trap. The optical trap was moved relative to the glass coverslip in order to separate the two strands of the dsDNA template and thus to displace

the nucleosome. Ligated downstream of the initial nucleosome was a long stretch of naked dsDNA.

(B) Configuration for helicase displacement of a nucleosome. The overall template configuration is the same as shown in (A). The fork was held under a constant tension (12 pN) below the force necessary for mechanical unzipping. T7 Helicase unwinding of the dsDNA resulted in an increase in the DNA extension.

A

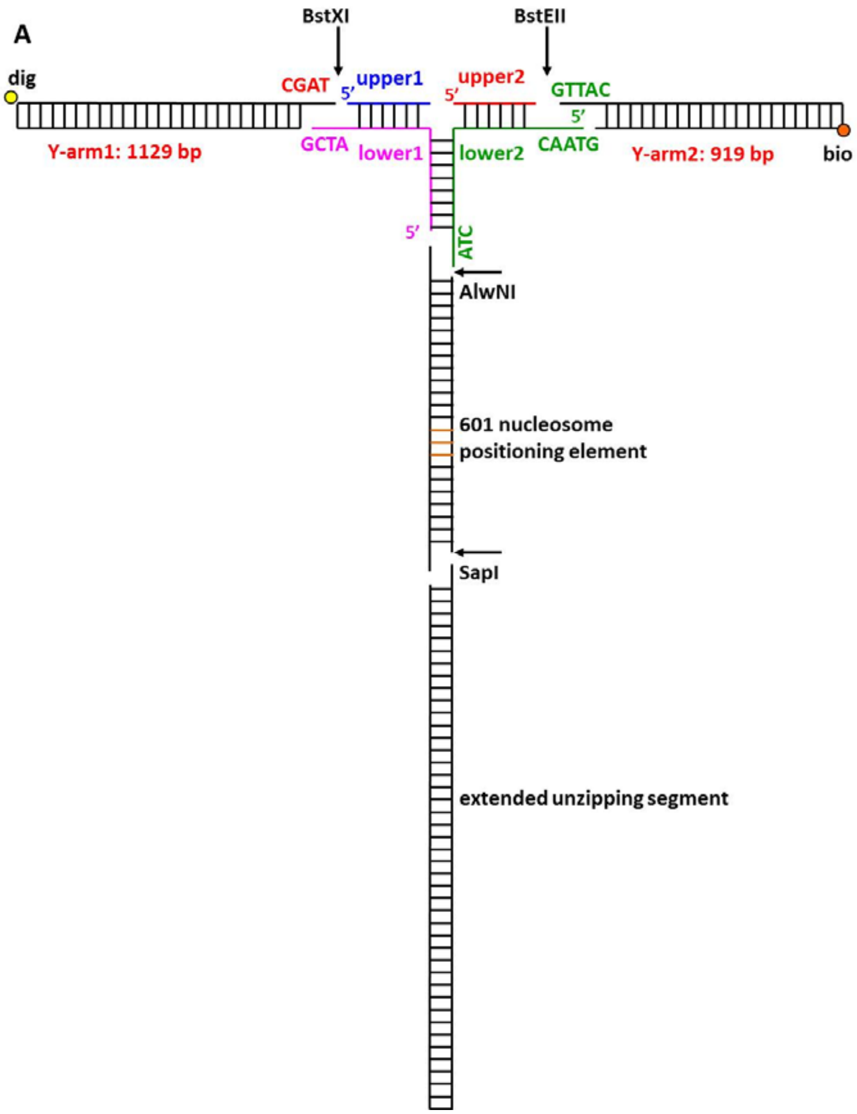
BstXI
BstEII
dig
CGAT
5′
upper1
5′
upper2
GTTAC
5′
GCTA
lower1
lower2
CAATG
Y-arm1: 1129 bp
Y-arm2: 919 bp
bio
5′
ATC
AlwNI
601 nucleosome
positioning element
SapI
extended unzipping segment

B

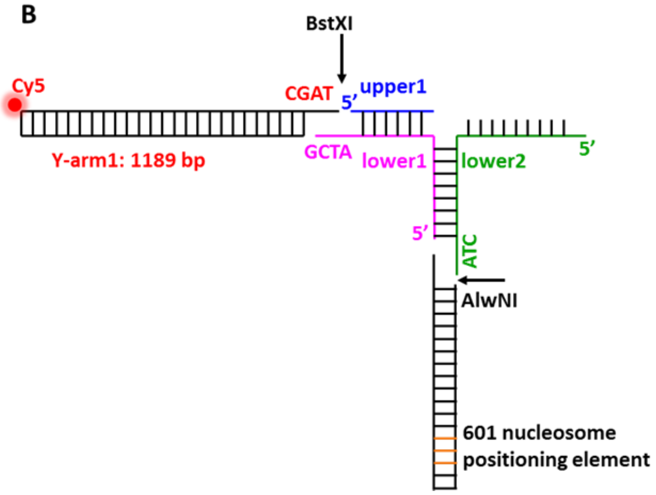
BstXI
Cy5
CGAT
5′
upper1
GCTA
lower1
lower2
5′
Y-arm1: 1189 bp
5′
ATC
AlwNI
601 nucleosome
positioning element

Supplementary Figure 2: DNA templates used in experiments.

(A) DNA templates for single molecule experiments were constructed as described in the methods[1]. The 601 nucleosome positioning element and extended unzipping sequence were ligated to the Y-arms in a single ligation step.

(B) Templates for bulk experiments were constructed using similar Y junction adapter oligos, except that upper2 was omitted, leaving 30 bp of ssDNA on the arm corresponding to the lagging strand to facilitate loading of T7 helicase. Arm1, which corresponds to the leading strand, was labeled with Cy5 at the 5' end, as shown. A 298 bp 601 nucleosome positioning element located at the distal end was ligated to the fork junction.

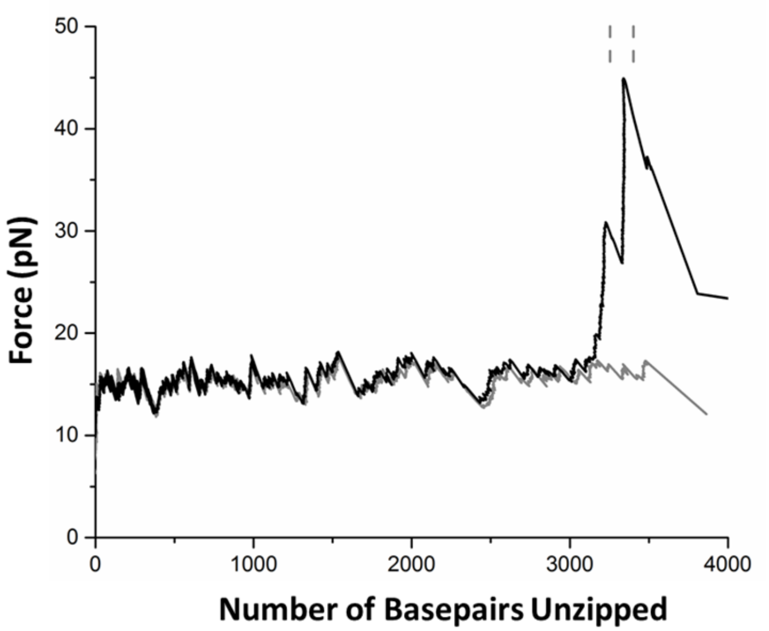

Supplementary Figure 3: Mapping of nucleosome position on a DNA template in the reverse direction as that described in Figure 1.

The nucleosomal DNA template mechanically unzipped in Figure 1 was constructed by ligation of a DNA template containing a single positioned nucleosome to a long naked DNA downstream. This design should ensure that the nucleosomes detected downstream of the initially nucleosome were a result of nucleosome transfer, and not due to additional nucleosomes pre-existing on the template. In order to verify this, we conducted a control experiment to mechanically unzip the same nucleosomal template as used in Figure 1 but in the reverse direction. The figure above shows a representative trace of this control experiment. Out of all the traces unzipped (*N* = 48), 92% showed a single nucleosomal force signature located within the expected 601 sequence (grey), and a complete absence of any force peaks above the baseline prior to the initial nucleosome. A few traces showed a force signature away from the 601 sequence, most likely due to over-assembly of the nucleosome used for this

control experiment. Note that the nucleosomal templates used in experiments for the main figures were slightly under-assembled (see Supplemental Figure 6 and Supplementary Table 2).

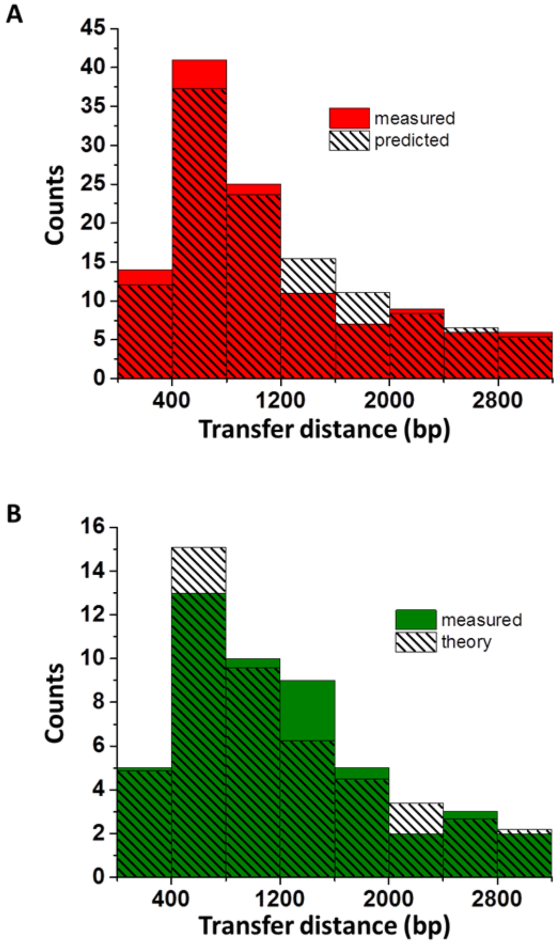

Supplementary Figure 4: Comparison of measured and predicted nucleosome transfer distances.

(A) The transfer distances from the mechanical displacement of a single nucleosome data (from Figure 1C) is overlaid with the predicted count calculated from the DNA-looping model, all binned in 400 bp increments. The Pearson test gives a reduced χ^2 of 0.53 with a *p*-value of 0.81 (see Methods for details).

(B) The transfer distances from the T7 helicase displacement of a single nucleosome data (from Figure 3C) is overlaid with the predicted count calculated from the DNA-looping model, all binned in 400 bp increments. The Pearson test gives a reduced χ^2 of 0.31 with a *p*-value of 0.95 (see Methods for details).

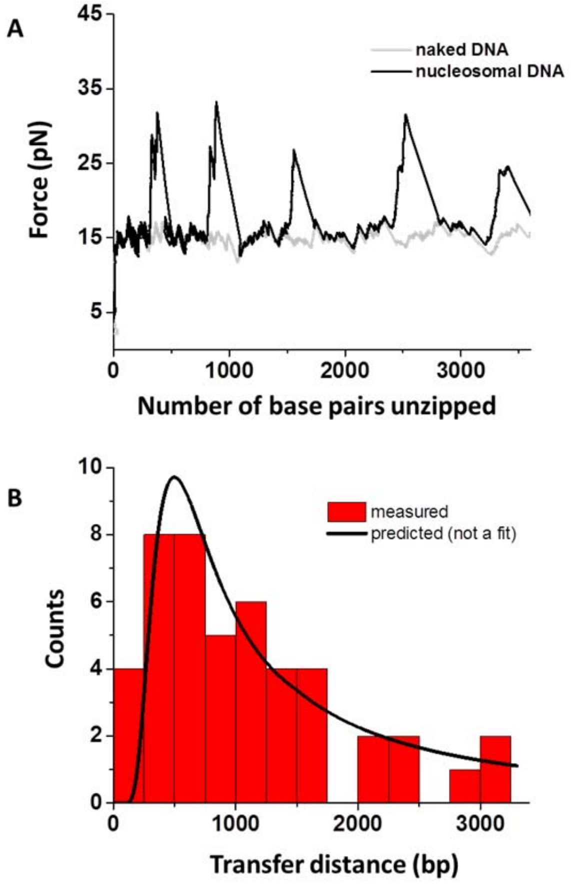

Supplementary Figure 5: Mechanical displacement of a nucleosome on a DNA template different from that described in Figure 1.

(A) Representative trace for mechanical displacement of a nucleosome on a different DNA template. The first 764 bp of the DNA template containing the 601 nucleosome positioning element was identical to that used for Figure 1. However, this segment was then ligated to a 2927-bp segment, which was identical to the 2987-bp segment used in Figure 1 except for the slightly shorter length, but had a reversed sequence (Methods). Therefore, if the peak position at 500-700 bp of transfer distance shown in Figure 1c were due to the DNA sequence favoring nucleosome association at those positions, then the peak position for this template would be shifted to 3000-3200 bp.

(B) The measured transfer distance distribution for this template shows no substantial differences from that of Figure 1c, again peaking at 500-700 bp. This suggests that DNA sequence is not a primary factor influencing our observed transfer distance distribution.

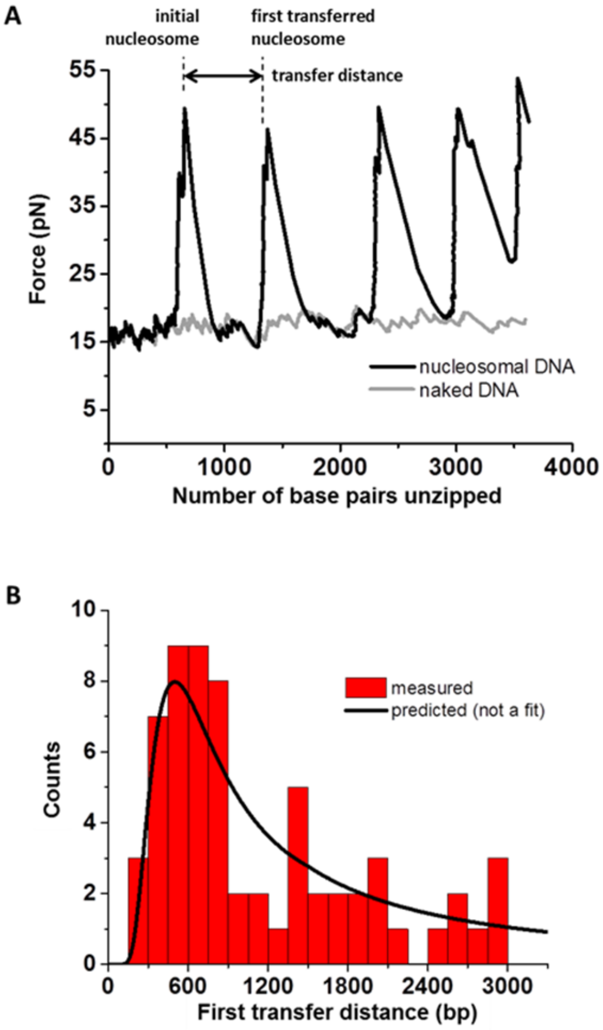

Supplementary Figure 6: Mechanical displacement of a nucleosome using a faster loading rate clamp.

(A) Representative trace for mechanical displacement of a nucleosome by unzipping DNA using a 100 pNs^{-1} loading rate clamp. The structures of the initial and transferred nucleosomes are consistent with those measured using a 10 pN s^{-1} loading rate clamp (Figure 1a), except for an increase in the peak forces.

(B) The measured transfer distance distribution at this faster unzipping rate and the prediction by the DNA loop formation model. This suggests that the rate at which the nucleosome is disrupted is slower than that of DNA loop formation, the latter being in the millisecond time scale [2]. There is, however, a slight increase in the nucleosome fraction for the first transfer event (Supplemental Table 2b).

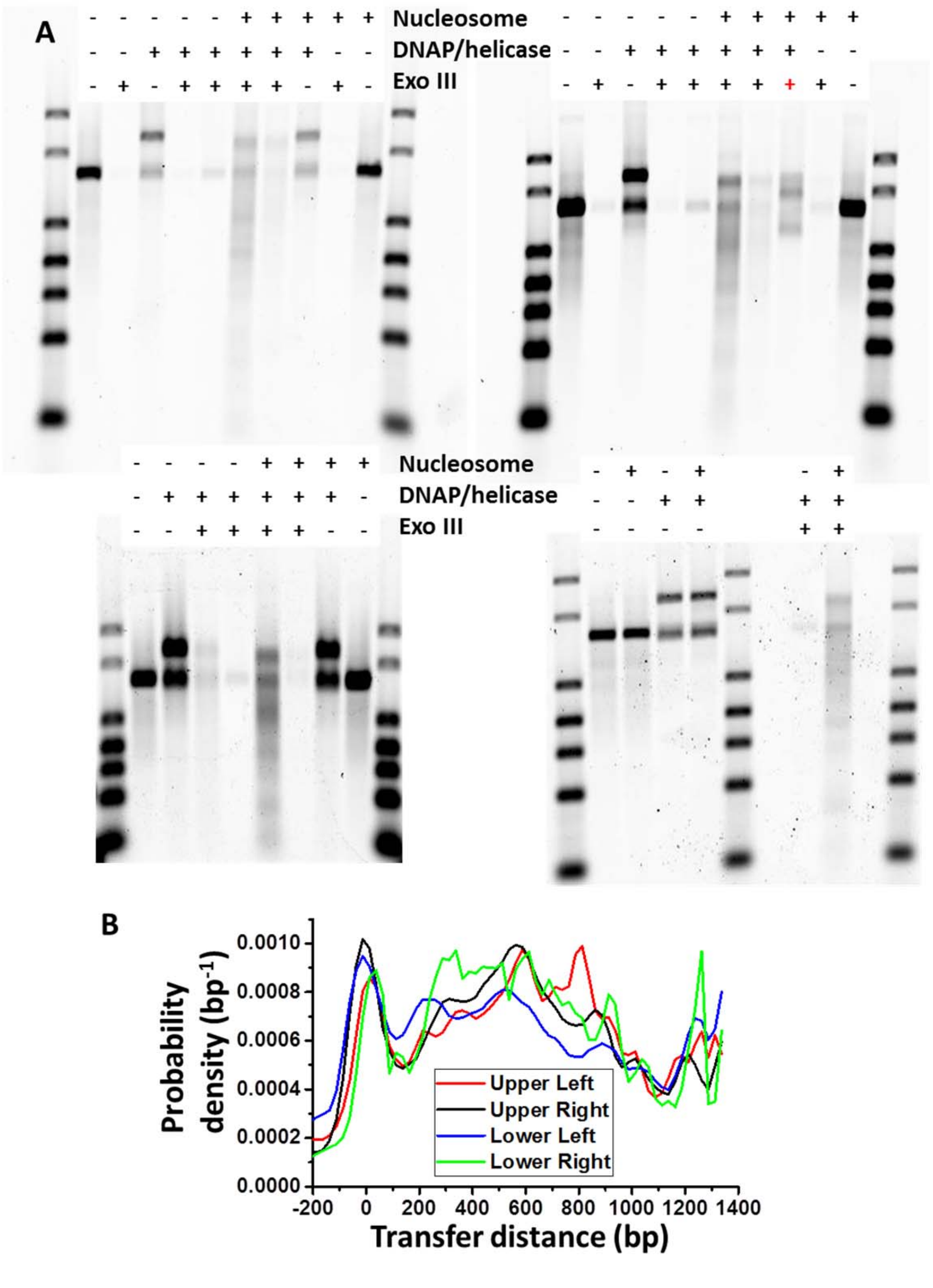

A
Nucleosome
DNAP/helicase
Exo III
B
Probability density (bp-1)
Transfer distance (bp)
Upper Left
Upper Right
Lower Left
Lower Right

Supplementary Figure 7: Additional replication gels for nucleosome transfer experiments**.**

(A) Additional replication gels for the nucleosome transfer experiment shown in Figure 3. The upper left panel shows the same replication gel as that for Figure 3, except that all lanes in the gel are included. Lanes shown in Figure 3 (from left to right) correspond to lanes 1, 2, 4, 11, 9, 6, and 7 in the full gel shown here. The remaining three gels are replicates. Unmarked lanes contain Cy5 labeled DNA ladders, with bands sized at 1788, 1390, 859, 649, 497, 332, and 125 bp, from top to bottom. Duplicate nucleosome/ replication/ exonuclease digestion lanes were loaded with equal (right) and 6-fold (left) amounts of DNA as the other lanes. Note that the red plus (+) over lane 8 in the upper right-hand gel indicates the accidental addition of exo-nuclease simultaneously with helicase and DNAP, this lane was excluded from all analysis.

(B) Nucleosome transfer distance distributions calculated from individual replicates shown in (A). The transfer distance distribution shown in Fig. 5A is the average of the four replicates.

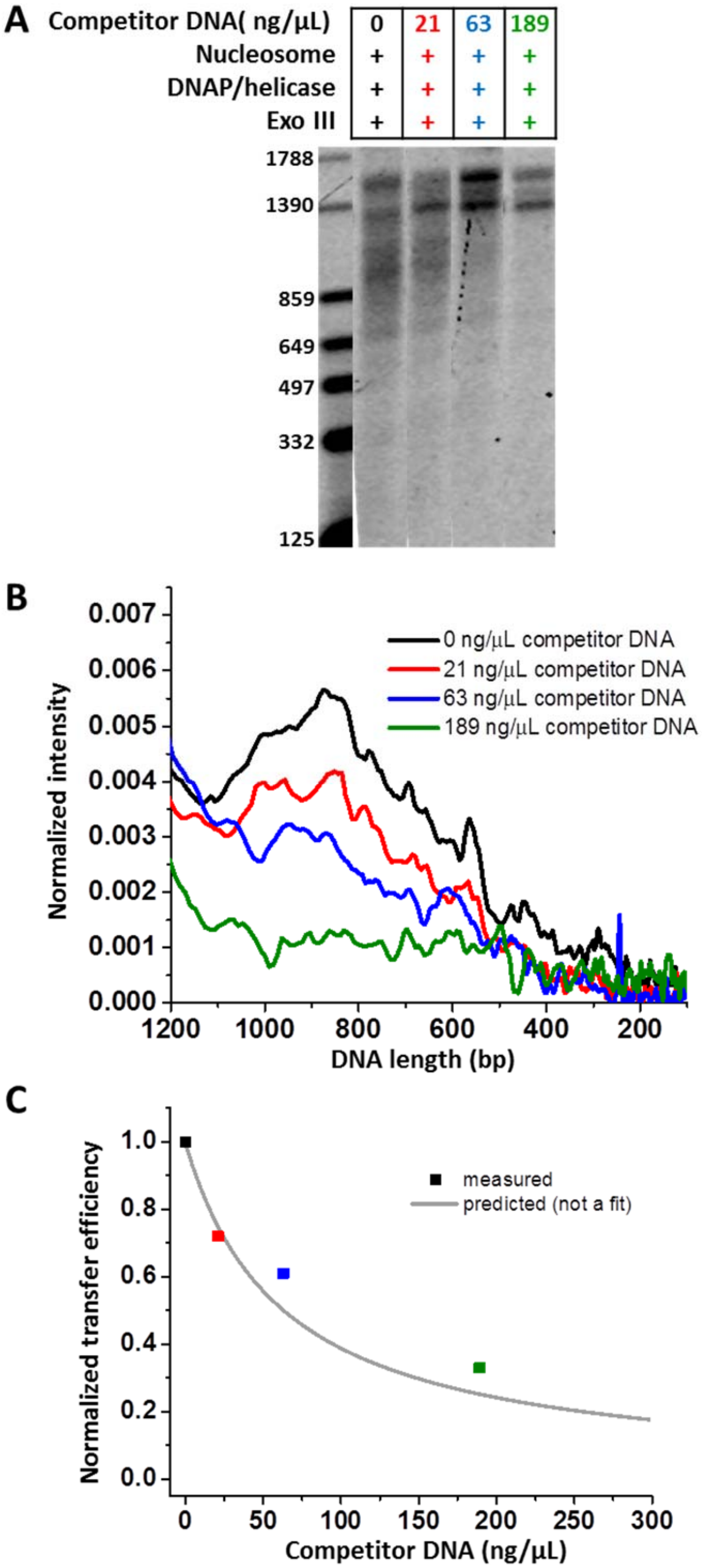
A
Competitor DNA(ng/μL)
0
21
63
189
Nucleosome
DNAP/helicase
Exo III
1788
1390
859
649
497
332
125
B
0 ng/μL competitor DNA
21 ng/μL competitor DNA
63 ng/μL competitor DNA
189 ng/μL competitor DNA
Normalized intensity
DNA length (bp)
C
measured
predicted (not a fit)
Normalized transfer efficiency
Competitor DNA (ng/μL)

Supplementary Figure 8: Replication gel for nucleosome transfer experiment in the presence of competitor DNA.

(A) Replication on a nucleosome template in the presence of increasing amounts of competitor DNA. The experiments were performed in the same fashion as those shown in Figure 3 except for the addition of competitor DNA. Exonuclease III digestion was used to footprint the transferred nucleosome, as described in the bulk replication section under Methods.

(B) Line scans of lanes in (A). Line scans were the background subtracted and normalized to the total amount of fully replicated DNA under each competitor DNA condition (data not shown).

(C) Normalized nucleosome transfer as a function of competitor DNA concentration. The measured normalized transfer frequency was calculated by integrating each line scan from 1200 bp to 100 bp (corresponding to 300 bp transfer distance to 1400 bp transfer distances) and normalizing it by that measured in the 0 ng μL^{-1} condition. The predicted transfer frequency was calculated as described in the competitor DNA section under Methods.

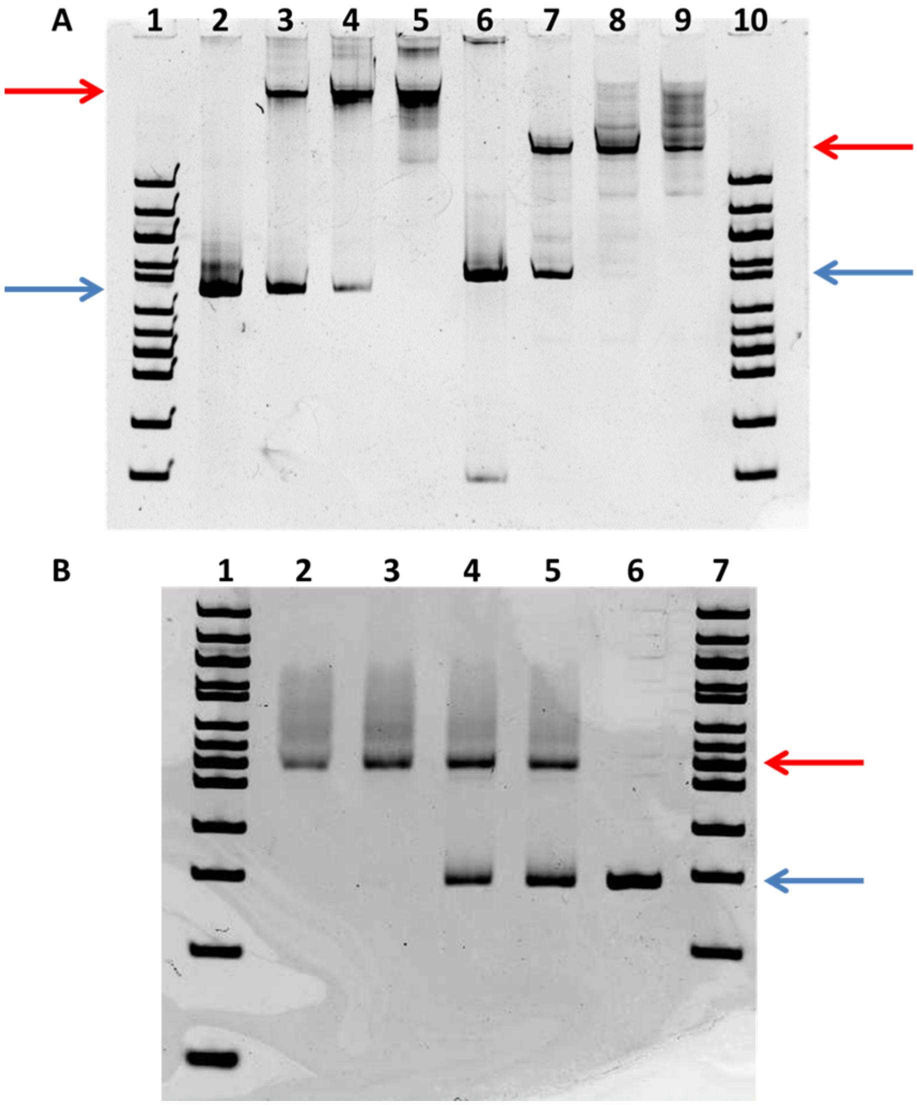

Supplementary Figure 9: Native gels of two representative nucleosome assemblies**.**

(A) 4% native acrylamide gel stained with ethidium bromide. Lanes 1 and 10 are 100 bp ladder (NEB). Lanes 2 and 6 are unassembled DNA segments used in mechanical disruption and helicase disruption experiments, respectively. Lanes 3-5 and 7-9 show assemblies with increasing (form left to right) amounts of histone octamer added during the assembly. Lanes 3 and 7 were used for data acquisition. Blue arrows indicate unassembled DNA. Red arrows indicate mono-nucleosomes.

(B) 4% native acrylamide gel stained with ethidium bromide. Lanes 1 and 7 are 100 bp ladder. Lane 6 shows unassembled DNA and lanes 2-5 are assemblies with decreasing (from left the right) amounts of histone octamer. Nucleosome assemblies in lanes 4 and 5 were used for the bulk replication assays.

**Experiments**	**DNA construct name**	**Sequence**
All optical trapping experiments	1 kb Arm 1	PCR from pRL574 using forward primer: 5'-/5digT/GTTGTAAAACGACGGCCAGTGAAT-3‘ and reverse primer: 5'-GATCCAGATCGTTGGTGAAC-3'
All optical trapping experiments	1 kb Arm 2	PCR from pBR322 using forward primer: 5'-CATTCCAACGATCTGGCGGC/iBiodT/GCGGTGCTC AAC GG-3' and reverse primer: 5'-ACGGTTACCAGCCTAGCCGGGTCCTCA-3'
All experiments	Upper Adapter 1	/5phos/GTCACCGATGCAGTACCGAGCTCATCCAATTCTA CATGCCGC
All experiments	Lower adapter 1	/5Phos/GCCTTGCACGTGATTACGAGATATCGATGATTGC GGCGGCATGTAGAATTGGATGA GCTCGGTACTGCATCG
All optical trapping experiments	Upper Adapter 2	CGTTACGTCATTCTATACACTGTACAGGTTACACGC
All experiments	Lower adapter 2	/5Phos/GTAACCTGTACAGTGTATAGAATGACGTAACGCG CAATCATCGATATCTCGTAATCACGTGCAAGGCCTA
Mechanical displacement of a nucleosome	764 bp nucleosome unzipping element	PCR from pLB601 (sequence available upon request) with forward primer: ACTCTTCGGGTAAAGTGCTGTATAACGCGC and reverse primer: CGCAGCTACTGTTCATACGACCAACCGCAGACAAGT
Mechanical and helicase displacement of a nucleosome	896 bp nucleosome unzipping element	PCR from pLB601 with forward primer: CGCTCTTCGGGTGAAACCGCATCTTTT and reverse primer: CGCAGCTACTGCATAAACTGAGACAGCTG
Mechanical and helicase displacement of a nucleosome	extended unzipping element	PCR from pLB601 with forward primer: /5FluorT/AACAGGATTAGCAGAGCGAGGTA and reverse primer: CGACCCGAAGAGCGCGCGTTATACAGCACTT
Mechanical displacement of a	reverse extended unzipping	PCR from pLB601 with forward primer: ACGCACCTGAAGAGCCCTAACTACGGCTACACTAG and

nucleosome	element	reverse primer: CGAATTCGAAGTCCAG
Replication gels	1189 bp Arm 1 Cy5	PCR from pLB601 with forward primer: /5Cy5/CAACGGTTACCGGACGCTC and reverse primer: CACACCACATCGCTGGCGTGTGTGTCCCG TC
Replication gels	289 bp nucleosome unzipping element	PCR from pMDW38 with forward primer: TGTGATGGACCCTATACGC and reverse primer: GGCCCAGCTACTGTAATACGACTCACTATAGGGC

Supplementary Table 1: Sequences for DNA constructs used in experiments.

This table shows a summary of all templates used. The exact sequences of all adapter oligonucleotides are given, as well as the primers and templates used in PCR reactions. All oligonucleotides were ordered from Integrated DNA Technologies (IDT) and use IDT's notation for modifications. Plasmids and their sequences are available upon request.

A

# of traces	Nucleosome	Tetrasome	Naked DNA
**Before disruption N=192**	$\mathbf{63.5^{+6.8}_{-7.2}}$ **%**	$\mathbf{6.7^{+4.6}_{-3.1}}$ **%**	$\mathbf{30.2^{+7.0}_{-6.4}}$ **%**
**First transfer N=122**	$\mathbf{67.2^{+8.2}_{-9.1}}$ **%**	$\mathbf{32.0^{+9.1}_{-8.2}}$**%**	$\mathbf{0.8^{+3.7}_{-0.8}}$ **%**
**Second transfer N=122**	$\mathbf{53.3^{+9.1}_{-9.3}}$ **%**	$\mathbf{42.6^{+9.3}_{-4.9}}$ **%**	$\mathbf{4.1^{+5.2}_{-2.8}}$ **%**

B

# of traces	Nucleosome	Tetrasome	Naked DNA
**Before disruption N=95**	$\mathbf{66.3^{+10.4}_{-9.4}}$ **%**	$\mathbf{7.4^{+7.2}_{-4.4}}$ **%**	$\mathbf{26.3^{+10.0}_{-8.5}}$ **%**
**First transfer N=63**	$\mathbf{81.0^{+8.8}_{-11.9}}$ **%**	$\mathbf{19.0^{+11.9}_{-8.8}}$ **%**	$\mathbf{0.0^{+5.7}_{-0.0}}$ **%**

Supplementary Table 2: Summary of the structures of transferred nucleosomes after mechanical displacement.

(A) Data from mechanical displacement of a nucleosome were analyzed as described in the Methods section and the number of traces for each type of nucleosome is included in these tables. Out of a total of 193 traces, 122 traces (63.2%) and 13 traces (6.7%) showed a force signature within the NPE consistent with that of a canonical nucleosome and a tetrasome respectively. The remaining 58 traces (30.1%) showed a force signature corresponding to naked DNA, indicative of a slight under-assembly of the nucleosomes. Only the traces with a nucleosome at the 601 positioning sequence were further analyzed (Figure 1C). For the first transfer event, out of 122 traces, 82 traces (67%) and 39 traces 32%) showed a force signature consistent with a canonical nucleosome or tetrasome,

respectively. Only one trace (0.8%) showed a positioned nucleosome without any subsequent transfer and was therefore not included in Figure 1C.

(B) For mechanical displacement of a nucleosome using a 100pN/s loading rate clamp, out of 95 traces, 63 (66.3%) traces showed a force signature consistent with that of a nucleosome at the initial position, 7 (7.4%) traces showed a signature indicative of a tetrasome, and the remaining 25 (26.3%) traces showed a force signature consistent with naked DNA. Only the traces with a nucleosome at the 601 positioning sequence were further analyzed. For the first transfer event, 51(81%) traces showed a nucleosome signature and 12 (19%) traces showed that of a tetrasome.

Supplementary References

1. Inman, J.T. et al. DNA Y structure: a versatile, multidimensional single molecule assay. *Nano Lett* **14**, 6475-80 (2014).
2. Hyeon, C. & Thirumalai, D. Kinetics of interior loop formation in semiflexible chains. *J Chem Phys* **124**, 104905 (2006).
